# GPCR-like signaling mediated by smoothened contributes to acquired chemoresistance through activating Gli

**DOI:** 10.1186/1476-4598-13-4

**Published:** 2014-01-07

**Authors:** Xia Zhan, Juan Wang, Yuan Liu, Yuanqiu Peng, Wenfu Tan

**Affiliations:** 1 Department of Pharmacology, School of Pharmacy, Fudan University, 826 Zhangheng Road, Shanghai 201203, P.R. China

**Keywords:** Smoothened, GPCR, JNK, Gli, Acquired chemoresistance

## Abstract

**Background:**

Smoothened (Smo), which possesses a structural similarity with classic G-protein coupled receptors (GPCR), is the most important molecular target in Hedgehog (Hh) signaling system for developing anticancer drugs; however, whether Smo may transmit GPCR-like signaling to activate the canonical transcriptional factor Gli of Hh signaling system and consequently to be involved in the Gli-dependent biological events remains controversial.

**Results:**

In this study, using the acquired chemoresistant cancer cell lines and their respective parental cells, we found that Smo may activate Gli through Gαi, Gβγ-JNK signaling axis, thereby promoting the Gli-dependent acquired chemoresistance. These observations were further complementarily strengthened by data obtained from chemosensitive cancer cells with artificially elevated Hh pathway activity.

**Conclusions:**

Hence, our data demonstrate that GPCR-like signaling mediated by Smo contributes to the acquired chemoresistance through activating the canonical Hh transcriptional factor Gli; therefore improving our knowledge of the nature of the signal transduction of Smo and the molecular mechanisms responsible for the acquired chemoresistance maintained by Hh pathway. Moreover, our data that JNK after activated by Smo-Gβγ signaling axis may stimulate the Gli activity and consequently promotes acquired chemoresistance expose a promising and potential target for developing anti-cancer drugs aimed at Hh pathway and for combating the acquired resistance raised by using of anti-cancer drugs targeting Smo.

## Introduction

The Hedgehog (Hh) signaling pathway plays critical roles for embryonic development and postnatal tissue homeostasis in organisms ranging from insects to mammals [1]. The activation Hh pathway is initiated by binding of the secreted Hh proteins, including Sonic, Indian and Desert Hedgehog (SHh, IHh and DHh, respectively) to the 12-transmembrane receptor Patched (Ptch), thereby liberating the Ptch-mediated inhibitory effect on Smoothened (Smo), a 7-pass transmembrane protein and a key component in the Hh signaling pathway. This subsequently causes the accumulation of Smo in the primary cilium and a series of consequent intracellular events, finally resulting in the activation of the canonical transcriptional factor Gli which consists of Gli1, Gli2 and Gli3 [2]. Analogous to other pathways active during embryonic development, inappropriate Hh pathway activity has been demonstrated to be critical for the initiation and progression of various kinds of tumors. Aberrant Hh pathway activity for tumors may occur either by mutations in key components of Hh pathway or by the production of Hh ligands in tumor cells in an autonomous and non-autonomous manner [3].

Given that the addiction of many types of tumors to aberrant Hh pathway activity, a variety of antagonists targeting Hh pathway have been developed for the treatment of cancers. Among them, the majority function as inhibitors of Hh pathway by targeting Smo, a critical component for canonical Hh pathway [4]. In this regard, dissecting the characteristics of signal transduction elicited from Smo is crucial and an area of intense investigation, as it may help us with the development of antagonists targeting Smo and its downstream effectors.

Smo, which possesses a structural similarity with classic G-protein coupled receptors (GPCR), has long been suggested to couple with heterotrimeric G proteins [5-7]. Indeed, it has been shown that Smo may interact with Gαi and subsequently acitivate the transcriptional activity of Gli (Ci in drosophila cells) in Drosophila Cl8 cells, Sf9 cells, and NIH3T3 cells, indicating the requirement of Gαi for the activation of Gli mediated by Smo [8,9]. However, this argument is challenged by the observations that *pertussis toxin* (PTX), which may ADP-ribosylate and consequently uncouple Gi from GPCR, fails to impact the Gli-dependent biological events such as chick neural tube patterning and some patterning events in zebrafish embryonic development [5,10]. On the other hand, after coupled to Gαi, Smo may exert a variety of biological activities independently of Gli, such as migration of murine embryonic fibroblasts, tubulogenesis of endothelial cells, and calcium spike activity of embryonic spinal cells [11-13]. Moreover, recent study indicate that Smo may contribute to the survival of diffuse large B-cell lymphoma cells by coupling to Gαi and Gα12 and subsequently activate NF-κB independently of Gli [14]. These studies suggest that the association between heterotrimeric Gαi proteins and Smo remains far from being fully understood, especially in the context of cancer biology. Meanwhile, in the case of canonical signal transduction of GPCR, ligand binding causes conformational changes in the structure of GPCRs, endowing them with abilities to function as a guanine nucleotide exchange factor (GEF). The exchange of GDP for GTP at the Gα subunit induces its dissociation from Gβγ dimmer [15]. To our knowledge, whether and how Gβγ, after dissociated from Gα subunit, may impact the Smo dependent Gli activity remains as well unclear.

Although great achievements have been made for the molecular-targeted anti-cancer drugs, traditional chemotherapy is still one of the most efficient approaches for treatments of cancers. Many studies have shown that Hh signaling pathway activity plays critical roles in maintaining the chemoresistant phenotype of acquired chemoresistant cancer cells [16-23]. In this study, utilizing well established acquired chemoresistant cancer cell lines and their respective parental ones, we provide a series of complementary evidences to show that Smo may promote acquired chemoresistance by activating Gli through Gαi and Gβγ-JNK signaling axis, therefore revealing that GPCR-like signaling elicited from Smo is involved in the canonical Hedgehog-Gli signaling pathway activation and the acquired chemoresistance.

## Materials and methods

### Drugs

Doxorubicin (Dox), Vincristine (VCR), Etoposide (VP16), Imatinib were purchased from Sigma-Aldrich (St. Louis, MO). The Hedgehog pathway antagonists cyclopamine (cyc), Robotnikinin (Robo) and GANT58 were obtained from Biovision (Milpitas, CA). The Gi antagonist Pertussis Toxin (PTX) was obtained from Invitrogen (Grand Island, NY). The JNK pathway antagonist TAT-TI-JIP was obtained from Calbiochem (Darmstadt, Germany). The agonist of Hh pathway SAG was obtained from Selleck Chemicals (Houston, TX).

### Cell culture

The K562 human chronic myelogenous leukemia cell line, KB human epidermoid carcinoma cell line, NIH-3 T3 mouse embryo fibroblast cells, and HEK293T human epithelial kidney cells were purchased from the American Type Culture Collection and cultured according to the manufacturer’s instructions. The Dox selected multidrug tolerant K562/A02 subline was obtained from the Institute of Hematology, Chinese Academy of Medical Sciences (Tianjin, China), which was routinely maintained in medium containing 200 ng/ml of Dox [24]. The VCR selected multidrug tolerant KB/VCR subline was obtained from Zhongshan University of Medical Sciences (Guangzhou, China) and was routinely maintained in medium containing 200 ng/ml of VCR [25]. Both resistant cells were authenticated by comparing their fold resistance with that of the parental cells and examining the expression levels of ABC transporters. All experiments using K562/A02, and KB/VCR cells were performed with cells growing in the absence of Dox or VCR for at least 5–7 days to avoid drug associated secondary effects.

### Plasmid constructions and l*entivirus*

8 × Gli-binding site luciferase reporter (8 × GBS-luciferase) and 8 × mutant Gli-binding site luciferase reporter (8 × GBS-luciferase mutant) plasmids were kindly provided by Dr. Hiroshi Sasaki. pRL-*Renilla* luciferase plasmid was purchased from Promega (Madison, WI). The mutant mouse plasmids SmoA1 (W539L) was generated from pEGFP-mSmo (a kind gift from Dr. Philip Beachy) using QuikChange Site-Directed Mutagenesis kit from Agilent (Santa Clara, CA) and confirmed by sequencing. The SmoA1 was next engineered into pLVX-EGFP-3FLAG-Puro lentivector. The pCDNA3 Flag MKK7-JNK1, pCDNA3 Flag JNK1(APF) and pCDNA3 Flag MKK7-JNK1(APF) plasmids were purchased from Addgene (Cambridge, MA) and confirmed by sequencing. These three plasmids were engineered into pLVX-EGFP-3FLAG-Puro lentivector. The PIRES2-ZsGreen1-Gβ1, PIRES2-ZsGreen1-Gγ2, and PIRES2-ZsGreen1-Gα transducin plasmids were purchased from Yrbio (Changsha, China) and were confirmed by sequencing. The Gα transducin (Gαt) plasmid was engineered into pLVX-EGFP-3FLAG-Puro lentivector.

Transient transfections were performed using Lipofectamine 2000 reagent (Invitrogen, Grand Island, NY) according to the manufacturer’s instructions. The viral stocks were prepared and infections were performed according to previously reported [26].

### Cell viability assay

The MTT assay was conducted as previously described to determine the sensitivity of cells to chemotherapeutic drugs [27].

### Reverse transcription PCR (RT-PCR) and quantitative RT-PCR (QT-PCR)

Total RNA was extracted from cells by RNAiso Plus Kit from TaKaRa (Dalian, China) as the instructions provided by the manufacturer and processed directly to cDNA by reverse transcription using SuperScript III kit (TaKaRa). Semi-quantitative PCR amplification was carried out using Stratagene mx3005p (Agilent Technologies). The quantitative PCR reactions were performed in triplicate with the SYBR-Green kit (TaKaRa) in iCycler iQ system (Bio-Rad; Hercules, CA). After reaction, the PCR products were subjected to electrophoresis to ensure the amplification from mRNA but not contaminated genomic DNA. The mRNA levels of interested genes were normalized to that of *TATA*. Primers for the genes tested were obtained from Invitrogen (Shanghai, China): *SHh*: 5′-CAAGCAGTTTATCCCCAATGTG-3′, 5′-TCACCCGCAGTTTCACTC-3′; *IHh*: 5′-TCAGCGATGTGCTCATTTTC-3′; 5′-AGCCGTAAAGAGCAGGTGAG-3′; *DHh*: 5′-TGCCGCTACTCTACAAGCAA-3′, 5′-GTTGTAGTTGGGCACGAGGT-3′; *Gli1*: 5′-GTGGGAAAGGTCTGGGATGT-3′, 5′-TGCGCCTGTCTCAGAGTAAAA-3′; *TATA*: 5′-ACCCTTCACCAATGACTCCTATG-3′, 5′-TGACTGCAGCAAATCGCTTGG-3′.

### Western blot analysis

Cells were lysed in lysis buffer (50 mM Tris, pH 7.4, 150 mM NaCl, 1% NP-40, 1 mM sodium vanadate, 1 mM PMSF, 1 mM DTT, 10 mg/ml of leupeptin and aprotinin) and subjected to immunoblot analysis. Primary antibodies against JNK, c-Jun, p-JNK, p-c-Jun, HA (Cell Signaling Technology; Beverly, MA), Flag, GAPDH (Santa Cruz Biotechnology; Santa Cruz, CA) were used for immunoblot analysis as standard procedure.

### Dual-luciferase reporter assay

Cells were seeded into 48-well plates. Twenty-four hours later, cells were cotransfected with luciferase expression constructs as indicated and *Renilla* luciferase using Lipofectamine 2000 (Invitrogen). Luciferase activities present in cellular lysates after indicated treatments were measured using a Dual-Luciferase reporter assay system from Promega (Madison, WI) according to the manufacturer’s instructions and a luminometer (Molecular Device; Sunnyvale, CA). The firefly luciferase values were normalized to *Renilla* values.

### Statistical analysis

Statistical differences were analyzed by the two-tailed Student’s t test and P < 0.05 was considered as significant. Asterisks denote statistical significance (*P < 0.05; **P < 0.01; and ***P < 0.001).

## Results

### Acquired chemoresistant cancer cells exhibit aberrant cell-autonomous Hh pathway activity

**Figure 1:**
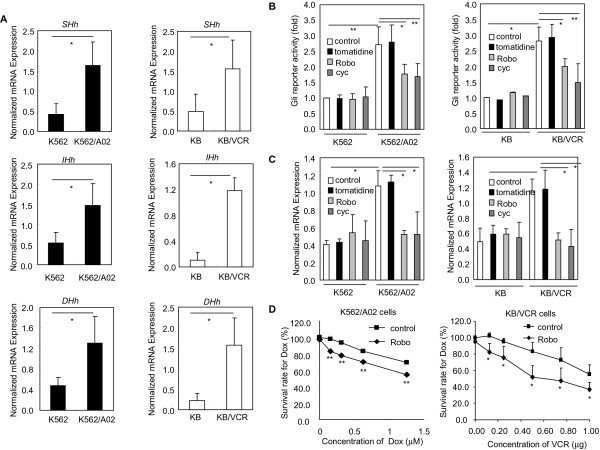
**Acquired chemoresistant cancer cells exhibit aberrant cell-autonomous Hh pathway activity.** Representative data from three independent experiments are expressed as mean ± s.d. **A**, Expressions of Hh pathway ligands *SHh*, *IHh* and *DHh* in acquired chemoresistant cancer cells K562/A02 and KB/VCR compared to their respective parental cancer cells K562 and KB respectively by QT-PCR analysis. **B**, Gli luciferase reporter activity in chemoresistant K562/A02 and KB/VCR cancer cells and their respective parental cancer cells K562 and KB determined by luciferase reporter assay. The cells (4 × 104) seeded into 48-well plates were transfected with Gli luciferase reporter and pRL-Renilla luciferase plasmids. After transfectinos for 24 h, the cells (around 100% confluency of adherent cells) in culture medium with 10% serum were further treated with tomatidine (10 μM), Robo (10 μM) and cyc (3 μM) for 24 h. **C**, Expressions of the Hh pathway target genes *Gli1* in acquired chemoresistant K562/A02 and KB/VCR cancer cells and their respective parental cancer cells K562 and KB. The cells (2 × 105) were seeded into 6-well plates. After 24 h, the cells (around 100% confluency of adherent cells) in normal culture conditions were treated with tomatidine (10 μM), Robo (10 μM) and cyc (3 μM) for 24 h were collected for examined by QT-PCR analysis. **D**, Treatment with the Robo circumvents the chemoresistance of K562/A02 cells and KB/VCR cells to Dox and VCR respectively. K562/A02 cells and KB/VCR cells were seeded into 96-well plates and incubated with Dox or VCR supplemented with or without various concentrations of Robo for 72 h.

The requirement of Hh pathway activity for maintaining the acquired chemoresistance indicates that acquired chemoresistant cancer cells may harbor aberrant Hh pathway activity via cell autonomous manner [3]. Elaborate verification of this argument is a prerequisite for dissecting the nature of the signal transduction from Smo to Gli in acquired chemoresistant cancer cells. In this regard, we first examined the expressions of ligands of Hh pathway in acquired chemoresistant cancer cells compared to their respective parental ones. Using two well established acquired chemoresistant cancer cells K562/A02, KB/VCR and their respective parental cells human chronic myelogenous leukemia cell line K562, human epidermoid carcinoma cell line KB, we found that the abundance of Hh ligands *SHh*, *IHh* and *DHh* were all obviously elevated when compared to their respective parental cancer cells as revealed by QT-PCR analysis (Figure 1A), suggesting the possibility of cell-autonomous Hh pathway activity harbored by acquired chemoresistant cancer cells. Next, we set out to evaluate whether the elevated production of Hh ligands correlates with the aberrant Hh pathway activity in acquired chemoresistant cancer cells using Gli-luciferase assay to rule out the non-cell autonomous Gli activation [3]. We observed that the chemoresistant cancer cells harbored aberrant Hh pathway activity relative to respective parental cells (Figure 1B). Meanwhile, treatment of acquired chemoresistant cancer cells with Robo and cyc, specific small molecular inhibitors targeting SHh and Smo, respectively [28,29], caused significant reductions of the aberrant Hh pathway activity in acquired chemoresistant cancer cells K562/A02 and KB/VCR, whereas both Robo and cyc did not affect Hh pathway activity in respective chemosensitive cells (Figure 1B). Moreover, tomatidine, a steriodal alkaloid structurally similar to cyc and lacking activity against Hh pathway [30], exhibited no effect on the Hh pathway activity in both chemoresistant and respective chemosensitive cancer cells (Figure 1B). These observations derived from Gli-luciferase reporter assay were faithfully recapitulated by QT-PCR analysis of the *Gli1* (Figure 1C), a transcriptional target of Hh pathway and served as readout of the Hh pathway activity. Considering that the chemoresistant cancer cells harbor aberrant cell-autonomous Hh pathway activity, it is conceivable that interference Hh pathway by targeting its ligands may sensitize acquired chemoresistant cancer cells to chemotherapy. Indeed, we found that exposure of chemoresistant cancer cells K562/A02, KB/VCR to Robo resulted in obviously sensitizing them to Dox and VCR, respectively (Figure 1D), while Robo treatment failed to impact the sensitivity of K562 and KB cells to Dox and VCR (data not shown). Hence, these data together clearly demonstrate that chemoresistant cancer cells harbor cell-autonomous Hh pathway activity.

### Gαi and Gβγ are involved in mediating the Hh pathway activity in chemoresistant cancer cells

**Figure 2:**
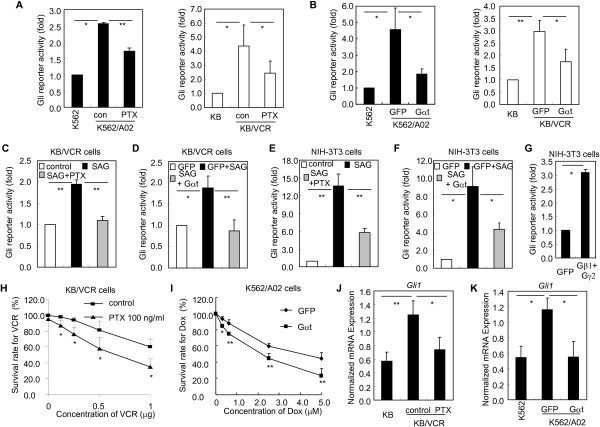
**Gαi and Gβγ are involved in mediating the Hh pathway activity in chemoresistant cancer cells.** The data are expressed as the means ± s.d. from three independent experiments. **A**, Cells as indicated were transiently transfected with Gli luciferase reporter and pRL-Renilla luciferase plasmids, followed by treatment with PTX (100 ng/ml) for 24 h. The data are expressed as fold induction relative to K562 cells. **B**, Cells as indicated were transiently transfected with Gli luciferase reporter and pRL-Renilla luciferase plasmids together with GFP or Gαt. The cells were harvested for dual luciferase reporter assay after transfection for 48 h. **C**, KB/VCR cells after transfected with Gli luciferase reporter and pRL-Renilla luciferase plasmids were treated with SAG (100 ng/ml) with or without PTX (100 ng/ml) in culture medium with 0.5% serum for 24 h. **D**, KB/VCR cells after transfected with Gli luciferase reporter and pRL-Renilla luciferase plasmids supplemented with GFP or Gαt were stimulated with SAG in culture medium with 0.5% serum for 24 h. **E**, NIH-3T3 cells were transfected with Gli luciferase reporter and pRL-Renilla luciferase plasmids, and were treated with SAG (100 ng/ml) with or without PTX (100 ng/ml) in culture medium with 0.5% serum for 24 h. **F**, NIH-3T3 cells after transfected with Gli luciferase reporter and pRL-Renilla luciferase plasmids supplemented with GFP or Gαt were stimulated with SAG in culture medium with 0.5% serum for 24 h and were collected for dual lucifearse assay. **G**, NIH-3T3 cells were transfected with Gli luciferase reporter and pRL-Renilla luciferase plasmids supplemented with GFP, and Gβ1 together with Gγ2 respectively. **H**-**I**, Interference with the functions of Gαi and Gβγ by treatment with PTX (**H**) and transfection with Gαt (**I**) increases the sensitivity of KB/VCR (**H**) and K562/A02 (**I**) to chemotherapeutic drugs. **J**-**K**, Interference with the functions of Gαi and Gβγ by treatment with PTX (**J**) and transfection with Gαt (**K**) reduces the *Gli1* expressions at mRNA level in KB/VCR cells and K562/A02 cells.

Having established that acquired chemoresistant cancer cells harbor cell-autonomous Hh pathway activation and represent ideal models for dissecting the signal transduction nature of Hh pathway, we then asked whether and how Smo may transmit GPCR-like signaling and consequently promote chemoresistance by activating Gli. Considering that Smo may couple to Gαi in Drosophila Cl8 cells, Sf9 cells, and NIH3T3 cells [8,9], we first set out to examine whether interference with Gαi may repress the Gli activity in chemoresistant cancer cells. Exposure of chemoresistant cancer cells to PTX, which may uncouple Gαi from receptor activation by ADP ribosylating Gαi [31], obviously suppressed the transcriptional activity of Gli in K562/A02 cells and KB/VCR cells as revealed by Gli-luciferase reporter assay (Figure 2A), indicating the involvement of Gαi in the Gli activation mediated by Smo in chemoresistant cancer cells. We next investigated the contribution of Gβγ to the activation of Gli in chemoresistant cancer cells by ectopic expression of Gαt, which may quench Gβγ upon dissociated from Gαi, thereby blocking the function of Gβγ [32]. As expected, ectopic expression of Gαt in K562/A02 cells and KB/VCR cells obviously suppressed the Gli-luciferase reporter activity (Figure 2B), thus indicating the participation of Gβγ in Gli activation. To further provide direct evidences for the argument that both Gαi and Gβγ are involved in mediating the signal from Smo to Gli, we asked whether intervention of Gαi and Gβγ may inhibit the Gli activation in response to SAG, a specific small molecular agonist of Smo [33]. SAG exposure provoked abundant Gli-luciferase reporter activity in KB/VCR cells ((Figure 2C,D), whereas PTX (Figure 2C) and Gαt (Figure 2D) obviously reduced the Gli-luciferase activity in response to SAG. These observations obtained by using KB/VCR cells were well recapitulated in NIH-3 T3 cells exposed to SAG (Figure 2E-F). Moreover, we observed that transfection of Gβ1 and Gγ2 plasmids into NIH-3 T3 cells remarkably stimulated the Gli-lucidferase reporter activity (Figure 2G); further demonstrating that Gαi and Gβγ upon dissociated from Gαi may transmit the signal from Smo to Gli. Taking these results together, we can conclude that Smo may couple to Gαi and both Gαi and Gβγ are involved in activating Gli mediated by Smo in chemoresistant cancer cells.

Next, we examined whether interference with Gαi and Gβγ may circumvent the chemoresistance of acquired chemoresistant cancer cells. We found that treatment of KB/VCR cells (Figure 2H) with PTX or ectopic expression of Gαt into K562/A02 cells (Figure 2I) by lenti-virus approach resulted in remarkably sensitizing the KB/VCR cells and K562/A02 cells to chemotherapeutic drugs VCR and Dox, respectively. Moreover, these reversals of acquired chemoresistance elicited by PTX and Gαt were accompanied by the repressions of Hh pathway activity in KB/VCR cells and K562/A02 cells as reflected by the reductions of transcript levels of *Gli1* (Figure 2J-K). Meanwhile, we observed that treatment of K562 and KB cells with PTX or transfection of Gαt did not increase the sensitivity of K562 and KB cells to chemotherapy (data not shown). Therefore, these data further demonstrate that Gαi and Gβγ are required for Smo-mediated Gli activation and consequently for Gli-dependent chemoresistance in acquired chemoresistant cancer cells.

### Gαi and Gβγ are required for the chemoresistance promoted by reconstituted Hh pathway activity in KB cells

**Figure 3:**
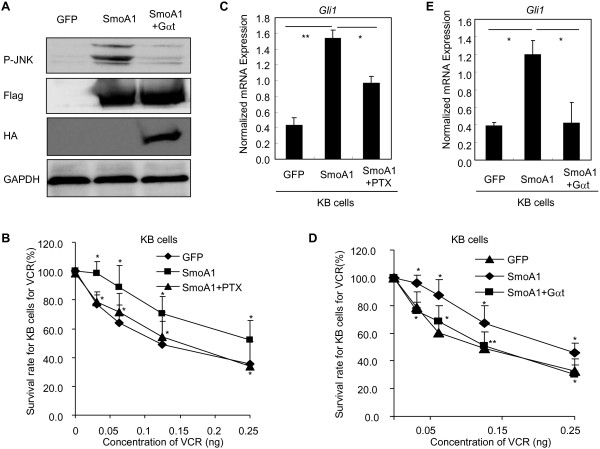
**Gαi and Gβγ are involved in the chemoresistance of KB cells with forced expression of SmoA1 by activating Gli.** KB cells were infected with lenti-virus harboring GFP, Flag-tagged SmoA1, and Flag-tagged SmoA1 plus Gαt with a HA tag. **A**, Western blot analysis of p-JNK, Flag tagged SmoA1 and HA tagged Gαt. **B**, SmoA1 causes KB cells insensitive to VCR, while PTX (100 ng/ml) treatment recovers the sensitivity of KB cells with forced expression of SmoA1 to VCR. Data are expressed as average ± s.d. from three separate experiments. **C**, SmoA1 enhances the expression of *Gli1* at mRNA level, while PTX treatment for 24 h abolishes this enhancement of *Gli1* expression at mRNA level. The results are expressed as the means ± s.d. from three to four independent experiments. **D**, SmoA1 causes KB cells insensitive to VCR, while co-expression of Gαt with SmoA1 by lenti-virus approach circumvents this tolerance to VCR. Data are expressed as average ± s.d. from three separate experiments. **E**, SmoA1 increases the expression of *Gli1* at mRNA level in KB cells compared to GFP cohort, while co-expression of Gαt with SmoA1 reduces the expression of *Gli1*. The results are expressed as the means ± s.d. from three to four independent experiments.

We next set out to provide complementary evidences for the notion that Smo may couple to Gαi and both Gαi and Gβγ may be involved in the Gli-dependent acquired chemorsistance mediated by Smo in chemoresistant cancer cells. Taking advantage of the lenti-virus approach, we constitutively activated the Hh pathway activity in chemosensitive cancer cells KB by ectopic expression of a Flag-tagged mouse mutant plasmid Smo (W539L, SmoA1), a frequent mutation in Smo which causes constitutive activation of Hh pathway in medulloblastoma cancers [34]. Ectopic expression of Flag-tagged SmoA1 in KB cells (Figure 3A) caused the KB cells insensitive to VCR treatment (Figure 3B), and concomitant activation of the Hh pathway activity in KB cells as judged by the increased expression of *Gli1* at mRNA level (Figure 3C). Of interest, PTX treatment or expression of HA-tagged Gαt by lenti-virus approach (Figure 3A) restored the sensitivity of KB cells with forced expression of SmoA1 to VCR (Figure 3B,D), paralleling the reductions of expression of *Gli1* at mRNA level (Figure 3C,E). Thus, these results together further strengthen that Gαi is coupled to Smo and both Gαi and Gβγ are involved in the Gli activation mediated by Smo and subsequently in maintaining the chemoresistance phenotype.

### Gβγ may promote Gli activity through JNK in chemoresistant cancer cells

**Figure 4:**
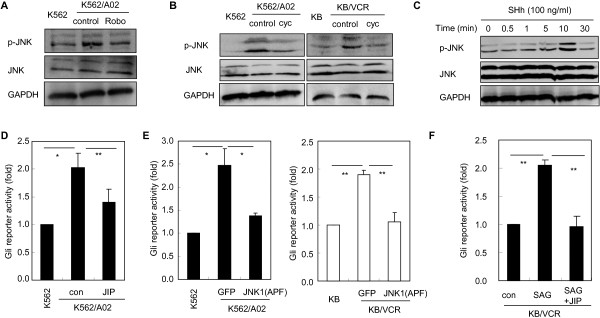
**JNK is involved in transmitting the signal from Hh to Gli in acquired chemoresistant cancer cells.** Values of the blots (p-JNK) relative to GAPDH quantified by Image J are statistical different (data not shown). **A**-**B**, Treatment with Hh inhibitors Robo **(A)** and cyc **(B)** suppresses the phosphorylation of JNK in acquired chemoresistant cancer cells. Blots are representative data from at least three independent experiments. **C**, Exposure of NIH-3 T3 cells to SHh causes phosphorylation of JNK. 293 T cells were treated with SHh (100 ng/ml) for different times as indicated, and were harvested for western blot analysis. GAPDH were used as a loading control. Blots are representative results from at least three separate experiments. **D**, K562/A02 cells and its parental K562 cells were transfected with Gli luciferase reporter and pRL-Renilla luciferase plasmids, and were treated with peptide JNK inhibitor JIP for 24 h. Data are mean ± s.d. from three separate experiments. **E**, Acquired chemoresistant cancer cells K562/A02, KB/VCR and their respective parental cells K562 and KB were transfected with Gli luciferase reporter and pRL-Renilla luciferase plasmids with GFP or Gαt. Data are mean ± s.d. from three separate experiments. **F**, KV/VCR cells were transfected with Gli luciferase reporter and pRL-Renilla luciferase plasmids, and were treated with SAG with or without JIP for 36 h. Data are mean ± s.d. from three separate experiments.

JNK is a well known downstream effector of Gβγ [35]. We then asked whether Gβγ, when released from Gαi after Smo activation, may activate Gli via JNK. To this end, we first tested whether inhibition of the Hh pathway may repress JNK activation in chemoresistant cancer cells. Exposure of K562/A02 cells with Robo (Figure 4A) or treatment of K562/A02 cells and KB/VCR cells with cyc (Figure 4B) led to reductions of the phosphorylation of JNK, indicating that Hh signaling may activate JNK in chemoresistant cancer cells. This was further supported by the observation that SHh significantly provoked JNK activation in 293 T cells, reaching a maximum at 10 min (Figure 4C). Next, we set out to determine the requirement of JNK activation for Gli activity in chemoresistant cancer cells. Inhibition of JNK in K562/A02 cells by JIP (Figure 4D), a peptide inhibitor specifically targeting JNK [36,37], or by transfection K562/A02 and KB/VCR cells (Figure 4E) with JNK1(APF), a plasmid of dominant negative mutant of JNK [38], abundantly impaired the Gli activity in chemoresistant cancer cells as judged by Gli-luciferase reporter assay, therefore suggesting that JNK is required for maintaining the cell-autonomous Gli activation in acquired chemoresistant cancer cells. This argument was further confirmed by the results that JIP obviously abolished the Gli activity provoked by SAG in KB/VCR cells (Figure 4F). Together, these findings suggest that JNK is involved in the cell-autonomous Gli activation in chemoresistant cancer cells.

**Figure 5:**
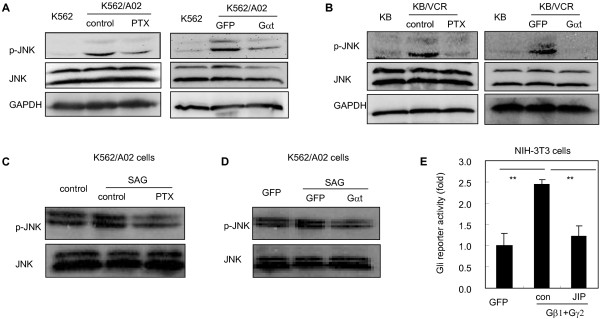
**Gβγ may transmit the signal from Smo to Gli via JNK.** Values of the blots (p-JNK) relative to JNK quantified by Image J are statistical different (data not shown). **A**-**B**, K562/A02 **(A)** and KB/VCR cells **(B)** were treated with PTX (100 ng/ml) or transfected with Gαt plasmids, and cells were harvested for western blot analysis. K562 and KB cells were used as negative control. Blots are representative results from at least three separate experiments. **C**, K562/A02 cells were pre-treated with PTX (100 ng/ml) for 1 h followed by exposure to SAG (100 nM) for 10 mins. Blots are representative results from at least three experiments. **D**, K562/A02 cells were transfected with GFP or Gαt plasmids, and were stimulated with SAG (100 nM) for 10 mins. The immunoblots are representative of three separate experiments with identical results. **E**, NIH-3 T3 cells transfected with Gli luciferase reporter and pRL-Renilla luciferase plasmids plus GFP or Gβ1γ2 plasmids were treated with JIP for 36 h, and were collected for dual luciferase reporter assay. The results are represented as fold induction relative to GFP cohort, and were represented as mean ± s.d. from three separate experiments.

We next set out to examine whether Gβγ may activate Gli though JNK in acquired chemoresistant cancer cells. We observed that treatment of chemoresistant cancer cells K562/A02 (Figure 5A) and KB/VCR (Figure 5B) with PTX or with transfection of Gαt resulted in decreasing the phosphorylation of JNK. Furthermore, PTX and Gαt remarkably abolished the phosphorylation of JNK in response to SAG in chemoresistant cancer cells K562/A02 (Figure 5C,D). Hence, these results suggest that Gβγ may mediate Gli activation elicited by Smo through JNK. This argument was further strengthened by the observation that JIP significantly inhibited the Gli-luciferase reporter activity provoked by transfection of Gβ1 and Gγ2 subunits into NIH-3T3 cells (Figure 5E). Collectively, our findings demonstrate that after released from Gαi, Gβγ may activate Gli via JNK in chemoresistant cancer cells.

### JNK activity is required for maintaining the chemoresistance maintained by Hh pathway

**Figure 6:**
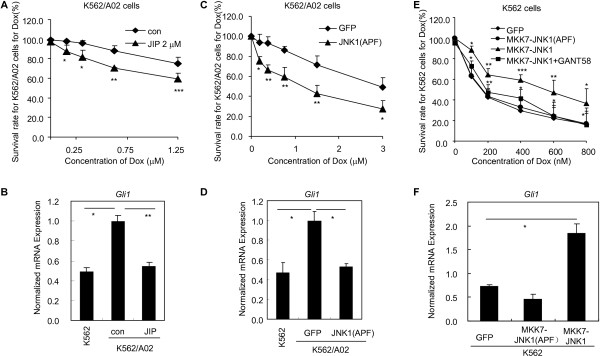
**JNK is involved in maintaining the chemoresistance of acquired chemoresistant cancer cells by activating Gli.** The data are expressed as the means ± s.d. from three independent experiments. **A**, Treatment with the peptide JNK inhibitor JIP circumvents the chemoresistance of K562/A02 cells to Dox. K562/A02 cells were seeded into 96-well plates, and incubated with vehicle control or Dox supplemented with or without JIP (2 μM) for 72 h. **B**, K562/A02 cells were treated with JIP (2 μM) for 24 h, and were collected for QT-PCR analysis of the expression of *Gli1*. **C**, Ectopic expression of JNK dominant negative mutant plasmid JNK1(APF) by lenti-virus approach enhances the sensitivity of K562/A02 cells to Dox. K562/A02 cells with stable expressions of JNK1(APF) and GFP were seeded into 96-well plates, and incubated with vehicle control or Dox for 72 h. **D**, K562/A02 cells with stable expressions of JNK1(APF) and GFP were collected for QT-PCR analysis of expression of *Gli1*. **E**, Activation of JNK in K562 cells by JNK constitutive mutant plasmid MKK7-JNK1 causes insensitivity of K562 cells to Dox, while inhibition of Gli activity by GANT58 recovers the sensitivity of K562 cells to Dox. K562 cells with forced expression of MKK7-JNK1, MKK7-JNK1(APF), GFP by lenti-virus approach were seeded in 96-well plates, and incubated with vehicle control or Dox supplemented with or without various GANT58 (10 μM) for 72 h. **F**, K562 cells with forced expression of MKK7-JNK1, MKK7-JNK1(APF), GFP by lenti-virus approach were harvested for QT-PCR analysis of *Gli1* expressions.

Having established that Gβγ may transduce the signaling from Smo to Gli via activating JNK in chemoresistant cancer cells, we next tested the biological relevance of Gli activation mediated by JNK to chemoresistance using acquired chemoresistant cancer cells. We saw that interference of JNK function with JIP or with forced expression of JNK dominant negative mutant JNK1a1 (APF) by lenti-virus approach significantly sensitized the K562/A02 cells to Dox (Figure 6A,C), concomitantly accompanying the reductions of the expressions of *Gli1* at mRNA level (Figure 6B,D). Given that JNK may promote chemoresistance through activating Gli, it is conceivable that artificial activation of JNK in chemosensitive cancer cells may result in Gli activation and subsequently chemoresistance. Indeed, taking advantage of the MKK7 and JNK1 fusion plasmid (MKK7-JNK1) engineered into a lenti-virus vector, which may activate JNK activity [39], we observed that artificial JNK activation rendered chemosensitive cancer cells K562 tolerant to Dox (Figure 6E), simultaneously increasing the Gli activity as reflected by QT-PCR analysis of the *Gli1* expression (Figure 6F); while the negative control MKK7-JNK1(APF) for MKK7-JNK1 did not impact either the sensitivity of K562 cells to Dox (Figure 6E) or the expression of *Gli1* at mRNA level (Figure 6F). Interestingly, GANT58, a small molecular antagonist specifically targeting Gli [40], restored the sensitivity of K562 cells with ectopic expression of MKK7-JNK1 to Dox (Figure 6E). Taken together, these results complementarily demonstrate that JNK may activate Gli in chemoresistant cancer cells, thereby maintaining the chemoresistance phenotype.

**Figure 7:**
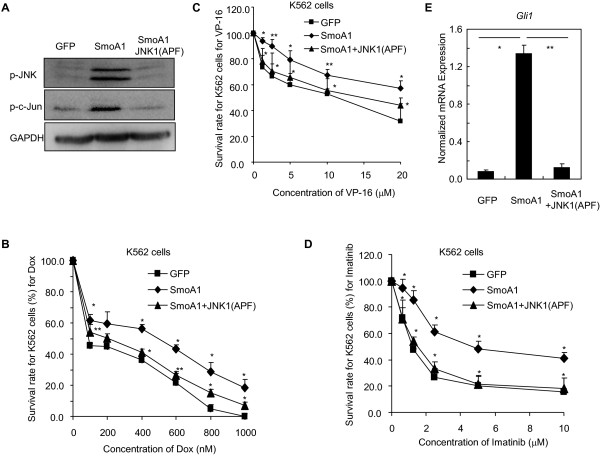
**JNK is involved in maintaining the chemoresistance of K562 cells with forced expression of SmoA1 by activating Gli. A**, K562 cells with forced expression of GFP, SmoA1 or SmoA1 plus JNK1(APF) by lenti-virus approach were collected for immunoblotting of phosphorylation of JNK and c-Jun. GAPDH were used as a loading control. **B**-**D**, Ectopic expression of SmoA1 causes K562 cells resistant to anti-cancer drugs Dox **(B)**, VP-16 **(C)**, and Imatinib **(D)**, while JNK dominant negative mutant JNK1(APF) abolishes these resistances. K562 cells with forced expressions of GFP, SmoA1 or SmoA1 plus JNK1(APF) by lenti-virus approach were seeded in 96 well plates, and were incubated with or without Dox, VP-16, and Imatinib for 72 h. Data are shown as mean ± s.d. from three separated experiments. **E**, K562 cells with forced expressions of GFP, SmoA1 or SmoA1 plus JNK1(APF) by lenti-virus approach were harvested for QT-PCR analysis of the expression of *Gli1* at mRNA level.

We next investigated whether the JNK activation is required for the chemoresistance promoted by ectopic expression of SmoA1. To this end, we artificially activated the Hh pathway activity using SmoA1 in K562 cells by lenti-virus approaches, like did in KB cells. Ectopic expression of SmoA1 in K562 cells resulted in obvious phosphorylations of JNK and its canonical downstream effector c-Jun, whereas JNK dominant negative mutant JNK1(APF) diminished these phosphorylations (Figure 7A), confirming the inhibitory effect of JNK1(APF) on the function of JNK. Similar to the observations obtained in KB cells, SmoA1 caused chemoresistance of K562 cells to Dox (Figure 7B), VP16 (Figure 7C) and BCR-ABL tyrosine kinase inhibitors Imatinib (Figure 7D), simultaneously accompanying increased Hh pathway activity as reflected by enhancement of *Gli1 mRNA* expression (Figure 7E). Moreover, JNK1 (APF) restored the sensitivity of the K562 cells with artificial elevated Hh pathway to Dox, VP16 and Imatinib (Figure 7C-D), concomitantly reducing the expression of Gli1 provoked by SmoA1 (Figure 7E). Collectively, our findings further confirm that JNK is involved in the chemoresistance mediated by Hh pathway and that after dissociation from Gαi initiated by Smo activation, Gβγ may stimulate the Gli activity through JNK and subsequently promote chemoresistance.

## Discussion

Hh signaling pathway has been shown to be critical for a variety of physiological and pathological conditions, such as embryonic patterning, maintenance of postnatal tissue homeostasis, as well as initiation and progression of cancers [41], whereas the molecular mechanisms responsible for its signaling transduction remain to be fully understood. Because of the structural homology with classical GPCRs, Smo has been suspected to have the ability to couple with heterotrimeric G proteins Gαi [5-7]. However, the contribution of Gαi to the Hh signaling transduction is quite controversial and unclear, especially in the cancer biology. Information available so far suggests that it is context-dependent and cell-type dependent for the ability of Smo coupling to Gαi and for the subsequent participation of Gli in the biological significance initiated by the interaction of Smo and Gαi [42,43]. In the present study, we provide complementary evidences to show that both Gαi and Gβγ are required for the Hh pathway activity and the subsequent acquired chemoresistance by activating its canonical transcriptional factor Gli, confirming the ability of Smo coupling to Gαi and the requirement of Gαi for the Gli-dependent biological significance in the context of acquired chemoresistance. Moreover, we found that Gβγ, after released from Gαi, are also be involved in the Gli activation and acquired chemoresistance through activating JNK. Indeed, by artificially increasing the Hh pathway activity in chemosensitive cancer cells, we determined that both Gαi and Gβγ-JNk signaling axis are required for the Gli activity and Gli-dependent acquired chemoresistance mediated by SmoA1. Our data that GPCR-like signaling mediated by Smo contributes to the acquired chemoresistance by activating Gli improve our interpretations of the underlying mechanisms for the acquired chemoresistance promoted by Hh pathway and help us with improving the chemotherapeutic efficiency by using Hh inhibitors [44]. Meanwhile, this study shed light on the understanding the nature of signaling transduction of Smo in cancer biology.

Deregulated Hh signaling has been implicated in a wide range of cancers, such as medulloblastoma, basal cell carcinoma, glioblastoma, leukemia, breast cancer, pancreatic cancer, prostate cancer, lung caner, colon cancer, to name a few [45]. Aberrant Hh pathway activity may result from gain-of-function and loss-of-function mutations in key components in Hh pathway, such as PTCH, Smo, and Sufu [3]. Hh pathway may as well be activated in tumors by overexpression of Hh ligands functioning in a cell-autonomous or non-cell autonomous manner. However, the activation of Hh pathway in tumor cells via a cell-autonomous manner is challenged by many controversial observations and remains to be fully elucidated [3], for example, the inability of mutationally activated Smo expressed in pancreatic epithelial to initiate pancreatic cancer [46]. In this study, we used the well established acquired chemoresistant cancer cell lines as an experimental model system for investigating the contribution of heterotrimeric G proteins and their downstream effectors to Gli activation mediated by Smo. Our data clearly demonstrate that acquired chemoresistant cancer cells harbor aberrant Hh pathway activity in a cell-autonomous manner, thus increasing our knowledge about the mechanisms behind Hh activation in cancers. On the other hand, many studies have shown the loss of Hh pathway activity in cancer cells possessing elevated Hh pathway activity after cultured *in vitro*[47-49], arguing against the use of *in vitro* cultured cancer cell lines for many kinds of investigations related to Hh pathway in cancer biology, ranging from dissecting molecular mechanisms underlying Hh signaling transduction to preclinical evaluation of Hh inhibitors. In this regard, our data that acquired chemoresistant cancer cells harbor aberrant Hh pathway activity in a cell-autonomous manner identify acquired chemoresistant cancer cell lines as potential and useful *in vitro* experimental model systems for investigations related to Hh pathway in cancer biology.

How does the Smo-coupled Gαi signaling link the transcriptional factor Gli in chemoresistant cancer cells? In the case of classical GPCR signaling transduction, the exchange of GDP for GTP at Gαi subunit results in the activation of Gαi, thereby repressing the adenyl cyclase and subsequently decreasing the conversion of ATP to cAMP. Reduced cAMP level implies downregulation of the activity of PKA [15]. Considering that PKA is the key determinant for proteasome proteolysis of Gli by phophorylating it at multiple sites [50,51], we can envision that Gαi after activated by Smo signaling may protect Gli from proteasome degradation by inhibiting the activity of PKA in chemoresistant cancer cells in despite of required further verifications. On the other hand, in the case of classic GPCR signaling transduction, the Gβγ dimmer after releasing from Gαi may stimulate a couple of downstream effectors, such as PKC, PI3K and JNK [35]. Data from other labs indicate that dissociated Gβγ dimmer initiated by Smo signaling may potentially promote the activation of Gli via PKC and PI3K in chemoresistant cancer cells [52-55]. However, in the present study, we provide complementary evidences showing that Smo may as well promote the activation of Gli via Gβγ-JNK signaling axis. Hence, our data together with that from other labs suggest that Smo utilizes the G protein signaling to its full potential for activating the transcriptional factor Gli.

JNK, a key member of the family of MAPKs, is also called stress activated protein kinase (SAPK) and can be activated by environmental and genototoxic stress and other extracellular stimulus [56]. JNK activation has also been linked to acquired chemoresistance by promotion of chemoresistance or by reversal of chemoresistance, relying on the duration and strength of the signaling [56,57]. Here, we show that JNK may function as a downstream effector of Gβγ for transmitting the signaling from Smo to Gli, thereby promoting the Gli-dependent acquired chemoresitance. Thus, this finding will help us with better understanding the role of JNK in acquired chemoresistance. Similar to ERK1/2, another critical member of MAPKs, JNK signaling is as well deregulated in many types of cancers [56,58]. However, the contribution of JNK in cancer development is complex and far from being fully elucidated, in other words, exhibiting context-specific and cell type-specific manner. JNK has been well known to confer the positive impact on proliferation and survival of cancer cells via its target AP1, a transcriptional factor composing Jun and Fos [59]. Of interest, data in our study imply that Gli represents a putative downstream target of JNK, thus facilitating our better interpretation of the molecular mechanisms responsible for promoting the development of cancers by JNK. Although inhibitors of membrane protein Smo have been approved for treatment of basal cell carcinoma, the early acquired resistance to such inhibitors proposes the need for additional downstream targets [60]. Hence, our data imply JNK as a new target for the treatment of the tumors with acquired resistance to Smo inhibitors. In this regard, how JNK promotes the activation of Gli is quite interesting, and is currently being investigated in our lab.

## Conclusions

In this study, we demonstrate that GPCR-like signaling mediated by Smo contributes to the acquired chemoresistance through activating the canonical Hh transcriptional factor Gli. Our data improve our knowledge of the nature of the signal transduction of Smo and the molecular mechanisms responsible for the acquired chemoresistance maintained by Hh pathway. Moreover, the findings that JNK after activated by Smo-Gβγ signaling axis may stimulate the Gli activity and consequently promote acquired chemoresistance expose a promising and potential target for developing anti-cancer drugs aimed at Hh pathway and for combating the acquired resistance raised by using of anti-cancer drugs targeting Smo.

## Competing interests

The authors declare that they have no competing interests.

## Authors’ contributions

XZ, JW, YL and YP conducted the experiments and were involved in data analysis. XZ helped with drafting the manuscript. WT designed the study, analyzed, and interpreted data, and drafted the manuscript. All authors read and approved the final manuscript.
